# Transmitted antiretroviral drug resistance in treatment naïve HIV-infected persons in London in 2011 to 2013

**DOI:** 10.7448/IAS.17.4.19747

**Published:** 2014-11-02

**Authors:** Katie McFaul, Charlotte Lim, Rachael Jones, David Asboe, Anton Pozniak, Sonali Sonecha, Marta Boffito, Nneka Nwokolo

**Affiliations:** 1 Chelsea and Westminster Hospital, London, UK; 2 West London Centre for Sexual Health, Chelsea and Westminster Hospital, London, UK; 3 Kobler Clinic, Chelsea and Westminster Hospital, London, UK

## Abstract

**Introduction:**

Previously published UK data on HIV transmitted drug resistance (TDR) shows that it ranges between 3 and 9.4% [1,2]. However, there are no recent data from populations where HIV transmission rates are increasing. The aim of this study was to assess the prevalence of TDR in untreated HIV-infected individuals attending three HIV specialist clinics under the HIV Directorate, Chelsea and Westminster Hospital and based throughout London – the Kobler Clinic, 56 Dean Street and West London Centre for Sexual Health.

**Methods:**

We included all patients with a HIV diagnosis, no history of antiretroviral therapy (ART) intake, attending one of the three clinics (Kobler (K), 56 Dean Street (DS) and West London (WL)), between 2011 and 2013 who started antiretrovirals. Reverse transcriptase (RT) and protease region sequencing was performed using Vircotype virtual phenotype resistance analysis. Drug resistance mutations were identified according to Stanford University HIV Drug Resistance Database (http://hivdb.stanford.edu/).

**Results:**

Among 1705 HIV-1-infected patients enrolled in the study, 1252 were males (919 were MSM), 107 were females and 346 had no gender recorded. Ethnicity was 51.1% white British/Irish/other, 6.1% African, 2.1% Caribbean, 2.8% Asian, 1.3% Indian/Pakistani/Bangladeshi, 4.2%, other, 3.2% not stated, and 29.2% unknown. 547 were from K (84.3% males, 48.3% MSM), 826 were from DS (84.3% males, 71.9% MSM), and 109 from WL (87.2% males, 56.0% MSM), 223 from other sites not specified. 77.5% (1321 of 1705) of patients had baseline viral resistance testing performed. Prevalence of primary resistance in those with a baseline viral resistance test was 13.5% overall: 19.3% in K, 14.9% in DS, and 14.7% in WL. The most common mutations detected were: NRTI: 184V, 215F, 41L; NNRTI 103N, 179D, 90I; PI 90M, 46I, and 82A. Among patients who tested with TDR, 79.1% had one single mutation, 18.7% and 2.2% exhibited dual or triple class-resistant viruses, respectively.

**Conclusions:**

This study across a large HIV Medicine Directorate reported an overall TDR prevalence which is higher than that previously published and with significant rates of NNRTI resistance at baseline.
